# *Lacticaseibacillus* spp.; Probiotic candidates from Palmyra palm sugar possesses antimicrobial and anti-biofilm activities against methicillin-resistant *Staphylococcus aureus*

**DOI:** 10.14202/vetworld.2022.299-308

**Published:** 2022-02-12

**Authors:** Watcharapong Mitsuwan, Phoomjai Sornsenee, Chonticha Romyasamit

**Affiliations:** 1 Akkhraratchakumari Veterinary College, Walailak University, Nakhon Si Thammarat, 80160, Thailand; 2 Center of Excellence in Innovation of Essential Oil, Walailak University, Nakhon Si Thammarat, 80160, Thailand; 3 One Health Research Center, Walailak University, Nakhon Si Thammarat, 80160, Thailand; 4 Department of Family and Preventive Medicine, Faculty of Medicine, Prince of Songkla University, Songkhla 90110, Thailand; 5 Department of Medical Technology, School of Allied Health Sciences, Walailak University, Nakhon Si Thammarat, 80160, Thailand

**Keywords:** antibacterial activity, *Lacticaseibacillus* spp, methicillin-resistant *Staphylococcus aureus*, Palmyra palm sugar, probiotics

## Abstract

**Background and Aim:**

Probiotics are beneficial microorganisms that play important roles by adhering to the gut and producing antimicrobial substances to inhibit pathogens. The objective of this study was to isolate and characterize the probiotic lactic acid bacteria (LAB) from Palmyra palm sugar, which can produce antimicrobial compounds against methicillin-resistant *Staphylococcus aureus* (MRSA), a new zoonotic and food-borne pathogens.

**Materials and Methods:**

Twenty-six LAB isolates were isolated from 30 Palmyra palm sugar samples. Three selected LAB were further characterized as probiotics. In addition, the antibacterial and anti-biofilm-forming activities of the probiotics’ culture supernatants against MRSA and food-borne pathogens were investigated. Finally, the selected probiotics were identified by aligning 16S rRNA sequences.

**Results:**

The three confirmed probiotics, WU 0904, WU 2302, and WU 2503, showed strong antibacterial activities against *S. aureus*, MRSA, *Escherichia coli* O157:H7, and *Listeria monocytogenes*, as measured by a broth microdilution assay. Among the LAB isolates, 82.22-86.58%, 91.83-96.06%, and 64.35-74.93% exhibited resistance to low pH, pancreatin treatment, and bile salts, respectively. It was found that 59.46% and 83.33% auto-aggregation was observed in 2 and 24 h, respectively. Moreover, 50.25-57.24% adhesion was detected after the incubation of the bacterial cells to Caco-2 cells.. Biofilm inhibition (82.81-87.24%) was detected after the treatment of MRSA with the culture supernatants, when compared with that to the control. By the alignment of 16S rRNA sequences, the isolate WU 2302 was identified as *Lacticaseibacillus* spp. with 98.82% homology when compared to the GenBank database.

**Conclusion:**

This study indicates that isolated probiotics can produce antimicrobial compounds against MRSA and food-borne pathogens. The obtained results strongly suggest that these probiotics are promising candidates for pharmaceutical products.

## Introduction

The emergence of antibiotic resistance of zoonotic and food-borne pathogens has raised concerns worldwide. *Staphylococcus aureus* is well known as a major cause of infection in humans and animals. The emergence of methicillin-resistant *S. aureus* (MRSA) is associated with significant morbidity and mortality in animals worldwide [1]. A previous study revealed that MRSA could be isolated from animals in slaughterhouses and food samples randomly tested in supermarkets [2]. In addition, MRSA has been identified as a new zoonotic pathogen that can be transmitted from animals to humans [2]. It has been reported that a new MRSA strain isolated from an animal farm had entered the human population, with subsequent studies supporting the possibility of farmworkers becoming infected [2,3]. The extensive range of diseases caused by *S. aureus* and MRSA is probably associated with virulence factors, including biofilms [4]. The propensity of *S. aureus*, including MRSA to form biofilms, causes significant morbidity and mortality in infected individuals [4]. The treatment of MRSA infections is becoming increasingly difficult due to the increased antibiotic resistance and biofilm production of these pathogens. There is thus a need for new therapeutic agents for treating MRSA-related diseases.

Probiotics are a group of beneficial microorganisms located in many parts of the human body, including the gut [5]. The genus *Lactobacillus* is defined as lactic acid bacteria (LAB) and belongs to the normal microbiota of the mucosa of humans and animals [6]. In the literature, 261 species of the genus *Lactobacillus* are reported, which differ at the phenotypic, ecological, and genotypic levels [7]. *Lacticaseibacillus paracasei*, also known as *Lactobacillus paracasei*, is part of the microbiota of the human and animal gut. This taxon plays an important role by adhering to the gastrointestinal tract [8], resulting in gastrointestinal tract stability and preventing intestinal infections [9]. Probiotics also produce antimicrobial peptides that inhibit several food-borne pathogens, including *Escherichia coli* O157 H7, *S. aureus*, MRSA, and *Salmonella* Typhi [6], as well as the biofilms produced by such pathogens [10]. In addition, probiotic colonization in the human gut reduces the gut area that can be colonized by pathogens [11]. Intestinal probiotics have been reported to alter gene expression in the mammalian gut mucosa, ultimately affecting the function of the gastrointestinal tract [11].

Palmyra palm sugar is a local beverage produced by Palmyra palm (*Borassus flabellifer* Linn.). The juice is popular in tropical Asian countries, including the south of Thailand [12]. Palmyra palm sugar is a natural sweetener derived only from the sap of Palmyra palm. It consists of several ingredients, including reducing sugars, alcohol, volatile compounds, vitamins, essential minerals, and phenolic compounds [13]. In addition, the juice and products from Palmyra palm sugar are an important source of natural bacteria, including LAB [6].

Furthermore, LAB isolated from fermented Palmyra palm sap are considered to be probiotics [6]. It has been reported that probiotic LAB isolated from fermented foods can be used in commercial fermented food products. In addition, probiotics have functional characteristics to inhibit the growth of important human pathogens [14]. According to FAO/WHO guidelines, probiotic bacteria must be safe for humans and animals. To select and characterize the probiotics, the bacteria have to survive under the conditions in the gastrointestinal tract, such as low pH, and the presence of pepsin and pancreatin [6]. Furthermore, they should play an important role by adhering to the gastrointestinal tract, including to colon epithelial cells, to protect the host from invasive pathogenic bacteria [6].

To the best of our knowledge, no studies on probiotics isolated from Palmyra palm sugar and their effects against MRSA have been reported in Asia, including Thailand. Therefore, the objectives of this study were to isolate probiotic bacteria from Palmyra palm sugar, which can produce antimicrobial compounds against MRSA and food-borne pathogens. LAB were isolated from Palmyra palm sugar and further characterized as probiotics. In addition, the antibacterial and anti-biofilm-forming activities of the culture supernatants of these probiotics were investigated against MRSA and food-borne pathogens. Finally, the selected probiotics were identified by aligning their 16S rRNA sequences.

## Materials and Methods

### Ethical approval

This article does not contain any studies with human participants or animals performed by any of the authors.

### Study period and location

The study was conducted from May 2021 to December 2021. A total of 30 Palmyra palm sugar samples was collected from local markets in Songkhla and Nakon Si Thammarat Provinces, Southern Thailand. Bacterial isolation, characterization, identification, and antimicrobial activity of the supernatant of the probiotics against the pathogens were carried out at Walailak University, Nakorn Si Thammarat, Thailand.

### Bacterial strains and growth conditions

All the bacteria were received from Bacterial laboratory, School of Allied Health Sciences, Walailak University, Thailand. Important pathogenic bacteria including *Acinetobacter baumannii* ATCC 3476, *E. coli* ATCC 25922, *E. coli* DMST 4112, *E. coli* O157:H7 DMST 2743, *Listeria monocytogenes* DMST 73303, *Salmonella* Enteritidis DMST 15676, *S. aureus* ATCC 25923, MRSA, and *Staphylococcus epidermidis* DMST 25505 were used for the experiments. The pathogens were cultured in brain heart infusion (BHI) broth (HiMedia, India), incubated at 37°C for 18 h, and stored in BHI broth containing 25% (v/v) glycerol at −80°C until further use.

### Isolation of LAB from Palmyra palm sugar

A total of 30 Palmyra palm sugar samples were collected from local markets in Songkhla and Nakon Si Thammarat Provinces, Southern Thailand. Isolation of LAB from Palmyra palm sugar (*B. flabellifer* L.) was carried out as described previously [6], with slight modification. Ten milliliter samples were serially diluted in 90 mL of Mann Rogosa Sharpe (MRS) broth (Difco, USA) and spread on MRS agar plates. The plates were incubated at 37°C for 48 h under aerobic culture conditions. A single colony of the bacteria on MRS agar was streaked on an MRS agar plate and incubated at 37°C for 24 h. The isolated bacteria were kept in MRS broth containing 25% (v/v) glycerol at −80°C until use.

### Inhibition of selected pathogens by the isolated LAB

To select LAB that can produce antibacterial compounds, agar well diffusion was used [6]. Briefly, pathogenic bacteria were cultured in BHI broth while LAB were cultured in MRS broth, incubated at 37°C for 18 h. The culture was adjusted to 0.5 McFarland standards. Then, 100 μL of the suspension was added and spread on MRS agar plates. Thereafter, 8-mm-diameter wells were cut on the surface of the agar using the back of a sterile blue tip. A total of 100 μL of the LAB cultures were loaded into each well. MRS broth medium and 3% hydrogen peroxide were included as negative and positive controls, respectively. The plates were incubated at 37°C for 24 h. Inhibition zones were measured using a Vernier caliper and recorded. Inhibition zones of 6-10 mm, 11-15 mm, and ≥16 mm were recorded as +, ++, and +++, respectively. In addition, the selected bacteria were tested for their hemolytic activities on blood agar. The experiments were performed in triplicate. Data are presented as mean±standard deviation.

### Identification of bacteria

The isolated bacteria were first classified using a standard bacterial Gram staining method and observed under a light microscope (Nikon, Japan). The catalase activity of the bacteria was determined using 3% hydrogen peroxide. Then, the selected bacteria were further identified by a molecular technique as described by Mitsuwan *et al*. [15] and confirmed by matrix-assisted laser desorption ionization (MALDI)-Biotyper (Bruker Daltonics, Karlsruhe, Germany), as per the manufacturer’s instructions. The bacteria were cultured on tryptic soy agar (Difco), incubated at 37°C for 24 h. Then, 35 bacterial colonies were collected and suspended in 50 mL of TE buffer. The samples were heated to 94°C for 5 min, after which they were placed on ice for 5 min and centrifuged at 10,000×g for 1 min. The bacteria were identified by amplifying their 16S rRNA genes using universal primers 27F: 5′-AGAGTTTGATCCTGGCTCAG-3′ and 14927F: 5′-GGTTACCTTGTTACGACTT-3′, as described previously [6] with minor modifications. Polymerase chain reaction (PCR) was performed in a total reaction volume of 20 μL, consisting of 1×PCR buffer (10 mM Tris-HCl, 50 mM KCl, pH 8.3), 2.5 mM MgCl_2_, 0.4 mM dNTPs, 1U of Taq DNA polymerase, 0.5 μM 16S rDNA primer pair, and 2 μL of DNA template. The PCR conditions were as follows: 5 min at 95°C; 30 cycles of 30 s at 95°C, 55 s at 60°C, and 72 s at 70°C; and final extension of 5 min at 72°C. The PCR products were held at 4°C until they were subjected to agarose gel electrophoresis. Sequencing was conducted using Applied Biosystems 3730xl (Macrogen, South Korea). Sequences were aligned with the NCBI database using the BLAST search tool to determine the sequence similarity [16].

A BLAST comparison of the 16S rRNA gene sequence was performed and the similarities to the type strains were calculated using NCBI’s database. Multiple alignments were performed with the program CLUSTAL_W [17] in the software BioEdit Sequence Alignment Editor version 7.2.5 (https://bioedit.software.informer.com/7.2/) [18]. A phylogenetic tree based on a multiple 16S rDNA alignment-based similarity matrix was constructed by the neighbor-joining method [19] using thesoftware package MEGA X (https://www.megasoftware.net/) [20]. Unknown bases were discarded from the analyses. Bootstrapping analysis was undertaken to test the statistical reliability of the topology of the neighbor-joining tree using 1000 bootstrap resampling of the data.

### Tolerance against low pH

The tolerance against low pH by the LAB was investigated as described previously by Romyasamit *et al*. [21]. Briefly, the bacteria were cultured in De Man, Rogosa, and Sharpe broth (MRS broth, HiMedia, India) and incubated at 37°C for 24 h. One milliliter of the culture was harvested and further centrifuged at 8,000×g for 5 min. The pellets were washed, resuspended in Phosphate Buffered Saline (PBS; pH 7.4), and adjusted to 0.5 McFarland standards. One milliliter of each sample was harvested and centrifuged at 8000×g for 5 min to collect cell pellets. Then, MRS broth at different pH levels of 2 and 3 was added and incubated at 37°C for 3 h. The viable bacteria were counted by spreading the bacterial suspension on MRS agar. The relative percentage of the survival rate was defined as described [6]:

Survival rate (%) = [Final (Log CFU/mL)/Initial (Log CFU/mL)]×100.

### Tolerance against pepsin, pancreatin, and bile salt

To characterize the probiotic strains, the microorganisms were tested for their ability to tolerate different enzymes/compounds in the intestinal tract, as described previously [21]. The enzymes/compounds included pepsin (3 g/L, pH 2), pancreatin (1 g/L, pH 8), and bile salt (3 g/L). The bacterial suspension was prepared as described above. One milliliter of each sample was harvested and centrifuged at 8000×g for 5 min to collect cell pellets. Then, the MRS broth containing pepsin, pancreatin, and bile salt was added and incubated at 37°C for 4 h. The viable bacteria were counted by spreading the bacterial suspension onMRS agar (HiMedia). The relative percentage of survival rate was defined as described [6]:

The relative percentage (%) = [Final (Log CFU/mL)/Initial (Log CFU/mL)]×100.

### Hydrophobicity and auto-aggregation

To characterize the effects of the probiotics, hydrophobicity and auto-aggregation were determined as described previously [6] with minor modifications. Bacterial suspensions grown overnight in MRS broth were harvested by centrifugation at 8000 rpm for 10 min. To determine the hydrophobicity, the bacteria were washed twice using PBS. The cells were then resuspended in PBS containing 0.1 mL of hexadecane (A0). Hydrophobicity was determined by measuring the optical density of the aqueous phase at 600 nm (A1). For auto-aggregation, the bacterial cells were adjusted to an OD_600nm_ value of 0.8-1. The suspension was then incubated at 37°C for 0, 2, and 24 h. Auto-aggregation at different time points was determined by measuring the optical density of the aqueous phase at 600 nm (A time). The values of hydrophobicity and auto-aggregation were calculated according to the following formulas as described [6]:

Hydrophobicity (%)=[(1-A_1_)/A_0_)×100

Auto-aggregation (%)=[1–(A_time_)/A_0_)×100]

### Antibiotic susceptibility test

To confirm the antibiotic susceptibility profiles, selected LAB cultures were carried out by the disk diffusion assay according to the Clinical and Laboratory Standards Institute (CLSI) guidelines [22]. Briefly, 35 single colonies of the bacteria grown on Muller-Hinton agar (MHA) (Difco, France) were then cultured in Muller-Hinton broth (Difco) for 4-6 h. The culture was adjusted to a density of 0.5 McFarland standard and spread on MHA. Several antibiotic discs (Oxoid, UK) containing ampicillin (10 μg), vancomycin (30 μg), gentamicin (10 μg), erythromycin (15 μg), clindamycin (2 μg), tetracycline (30 μg), kanamycin (30 μg), chloramphenicol (30 μg), and streptomycin (10 μg) were tested. The plates were incubated at 37°C for 18 h. The inhibition zone of each antibiotic was measured and compared to the standard values in accordance with CLSI guidelines [22].

### Adhesion of the selected probiotics to Caco-2 cell line

To investigate the adhesion of the probiotics, human colorectal adenocarcinoma Caco-2 cells were used to create a model of the intestinal mucosa. The experiment was carried out as described previously [6] with some modifications. The cell line was cultured in Dulbecco’s Modified Eagle Medium (DMEM, Thermo Fisher Scientific, USA) supplemented with 10% fetal bovine serum (Gibco, USA), 3 mM L-glutamine, and antibiotics (50 μg/mL streptomycin-penicillin, 50 μg/mL gentamicin, and 1.25 μg/mL amphotericin B) (Thermo Fisher Scientific). Caco-2 cells were added to 24-well plates and incubated at 37°C in 5% CO_2_ to form a monolayer (80-90% confluence). Then, the bacterial cells (1×10^8^ CFU/mL) were added to the colon cells and incubated at 37°C for 2 h under 5% CO_2_. Unbound bacteria were removed by washing with sterile PBS 4 times. Subsequently, 200 μL of trypsin (2.5%, w/v) was added to break down the cell monolayer. A complete medium (800 μL) was added to recover the adherent bacteria. The bacterial samples were serially diluted and plated on MRS agar to count the adherent bacteria to the Caco-2 cell lines. Adhesion ability (%) was calculated with the following formula as described [6]:

% Adhesion ability = (V_1_×100)/V_0_

where V_0_ is the initial viable count and V_1_ is the viable count adhered to the Caco-2 cells after incubation.

### Effects of probiotic supernatants on biofilm formation of *S. aureus*

The effects of probiotic supernatants on the biofilm formation of *S. aureus* ATCC 25923 and MRSA were investigated using a crystal violet assay, in accordance with a modified version of the procedure of Mitsuwan *et al*. [23]. Probiotic bacteria including WU 0904, WU 2302, and WU 2502 were cultured in MRS broth and incubated at 37^o^C for 24 h. The culture supernatants of the probiotics were collected and added to 96-well plates. *S. aureus* ATCC 25923 and MRSA were cultured in tryptic soy broth (Difco) supplemented with 0.5% (w/v) glucose. The inoculum of the pathogens (1×10^6^ CFU/mL) was added to the 96-well plates containing the supernatants of probiotics. The culture medium alone was used as a negative control. The plates were incubated at 37^o^C for 24 h. The wells were washed twice with PBS and stained with 1% crystal violet for 30 min. The plates were washed with distilled water. The biofilm was dissolved with 100% dimethyl sulfoxide. Inhibitory activity was investigated by quantifying the biofilm formation at an optical density of 570 nm. The relative percentage of the biofilm inhibition was defined as described [23]:

The relative percentage of the biofilm inhibition (%)=100–[(570_treatment_/A570_control_)×100].

### Statistical analysis

The experiments were carried out in triplicate. The data were recorded and entered using statistical package software (SPSS Inc. Chicago, IL, USA). The results are presented as mean±standard deviation. Statistical analysis was performed by a two-tailed unpaired Student’s t-test. In all analyzes, p<0.05 was considered statistically significant.

## Results

### Isolation of LAB from Palmyra palm sugar

Among 30 samples of Palmyra palm sugar, 26 isolates of LAB were identified as Gram-positive, non-endospore-forming, and catalase-negative bacteria.

### Antimicrobial activity of LAB against pathogens

The antimicrobial activity of LAB isolated from Palmyra palm sugar against pathogens was preliminarily investigated by agar well diffusion assay. The results demonstrated that the supernatant of the bacterial cultures inhibited the growth of the tested pathogens (Table-1). It was highlighted that the isolates including WU 0904, WU 2302, and WU 2503 showed strong antibacterial activities against *E. coli*, *L. monocytogenes*, *S. aureus*, and MRSA (Table-1), which were selected for further studies. In addition, the non-hemolytic activity of all selected bacteria was observed (data not shown).

**Table 1:** Antimicrobial activity of the selected lactic acid bacteria isolated from Palmyra palm sugar against zoonotic and food-borne pathogens.

Bacteria	WU0904	WU2302	WU2502
*Escherichia coli* O157:H7 DMST 2743	++	+++	++
*Escherichia coli* DMST 4212	+++	+++	+++
*Listeria monocytogenes* DMST 17303	+++	+++	++
*Staphylococcus aureus* ATCC	+++	+++	++
Methicillin-resistant *Staphylococcus aureus*	+++	+++	++
*Staphylococcus epidermidis* DMST 25505	++	++	++
*Salmonella* Enteritidis DMST 15676	++	+++	+
*Acinetobacter baumannii* ATCC 3476	++	++	+

+=Inhibition zone: 6-10 mm, ++=Inhibition zone: 11-15 mm, and +++=Inhibition zone>16 mm

### Survival of probiotics under gastrointestinal tract conditions

To characterize the LAB as probiotics, the survival of the bacteria under conditions matching those in the gastrointestinal tract was investigated. All isolates showed relatively strong resistance to pancreatin (Table-2). Interestingly, 91.83-96.06% of the isolates showed resistance to enzyme treatment, 82.22-86.58% showed resistance to pH 3, and 64.35-74.93% showed resistance to treatment with bile salts. However, the isolates did not survive treatment with pepsin or pH 2.

**Table 2:** Survival rate (%) of the selected lactic acid bacteria isolated from Palmyra palm sugar under gastrointestinal tract (GIT) conditions.

GIT conditions	Lactic acid bacteria		

	WU0904	WU2302	WU2502
pH=2	ND	ND	ND
pH=3	82.22±5.02	88.05±1.75	86.58±1.14
Pepsin	ND	ND	ND
Pancreatin	96.06±0.73	93.37±0.23	91.83±0.08
Bile salts	70.97±2.51	64.35±5.74	74.93±0.48

ND=Not detectable.

### Auto-aggregation assay

**Figure-1:**
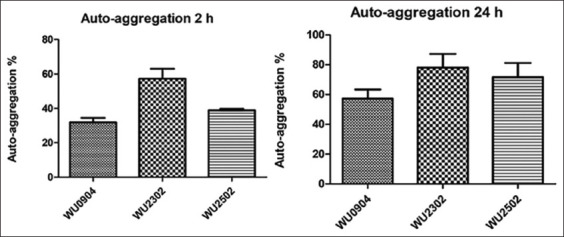
Auto-aggregation (%) of the selected lactic acid bacteria isolated from Palmyra palm sugar. The suspension of the bacteria was incubated at 37°C for 0, 2, and 24 h. Auto-aggregation at different time points was determined by the optical density of the aqueous phase at 600 nm. The data are presented as mean ± SD.

An Auto-aggregation assay was carried out to characterize the LAB properties as probiotics. The results demonstrated that the ability of the isolates to auto-aggregate increased with increasing incubation period (Figure-1). The isolate WU 2302 showed the highest auto-aggregation. Furthermore, 59.46% and 83.33% of the isolates were auto-aggregated at 2 and 24 h, respectively. At 24 h, isolates WU 0904 and WU 2502 showed rates of auto-aggregation of 54.55% and 70.59%, respectively.

### Cell surface hydrophobicity, ability of the isolates to adhere to Caco-2 cells, and antibiotic susceptibility profiles

**Figure-2:**
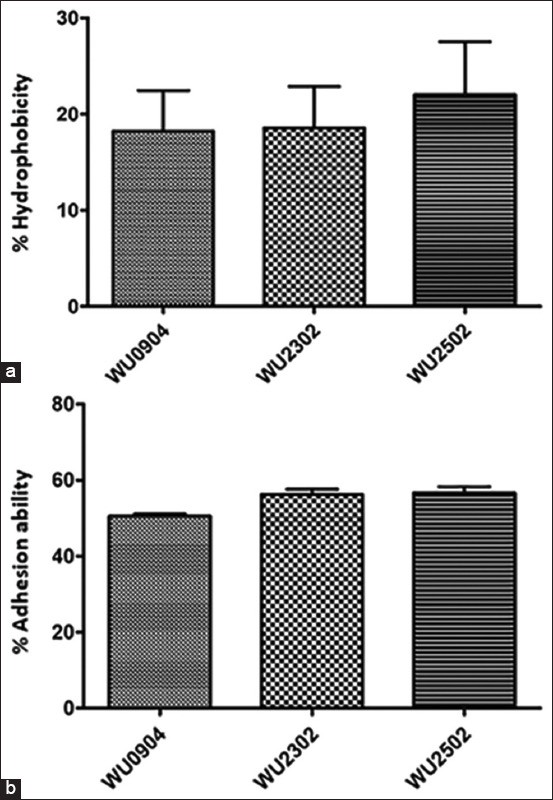
Percentage of hydrophobicity (a) and adhesion (b) of the selected lactic acid bacteria isolated from Palmyra palm sugar to Caco-2 cells. The data are presented as mean ± SD.

**Figure-3:**
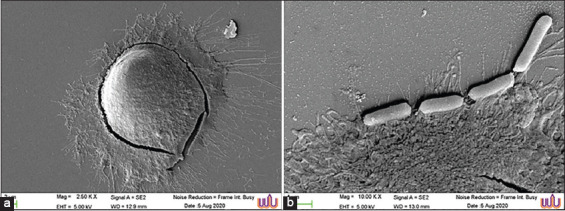
Scanning electron microscope (SEM) analysis of Caco-2 cells where the probiotic isolate adheres to the surface of Caco-2 cells. a; Untreated Caco-2 cells as control, b; adhesion of the probiotics to Caco-2 cells. Magnification: a = 2500×, b = 10,000×

Cell surface hydrophobicity was investigated to characterize the properties of the LAB as probiotics. Among the isolates, 17.65-21.18% exhibited hydrophobicity of the cells (Figure-2a). In addition, to characterize LAB as probiotics, the ability of the isolates to adhere to Caco-2 cells was investigated as an *in vitro* model of the intestinal epithelial barrier. The results demonstrated a rate of adhesion to Caco-2 cells of 50.25-57.24% after incubation of the bacterial cells with Caco-2 cells (Figure-2b). The morphology of bacterial cell free Caco-2 cells is shown in Figure-3a. The adhesion of probiotics to the surface of colon cells was also elucidated, as shown in Figure-3b. In addition, antibiotic susceptibility profiles of the selected three probiotics isolated from Palmyra palm sugar are presented in Table-3.

**Table 3:** Antibiotic susceptibility profiles of the selected probiotics isolated from Palmyra palm sugar.

Isolated strains	Ampicillin (10 μg)	Vancomycin (30 μg)	Gentamicin (10 μg)	thromycin (15 μg)	Clindamycn (2 μg)	Tetracycline (30 μg)	Kanamycin (30 μg)	Chloramphenicol (30 μg)	Streptomycin (10 μg)
WU0904	R	R	R	S	S	S	R	S	R
WU2302	R	R	R	S	S	S	R	S	R
WU2502	R	R	R	S	S	S	R	S	R

R=Resistant and, I=Intermediate, S=Susceptible.

### Inhibition of biofilm formation

**Figure-4:**
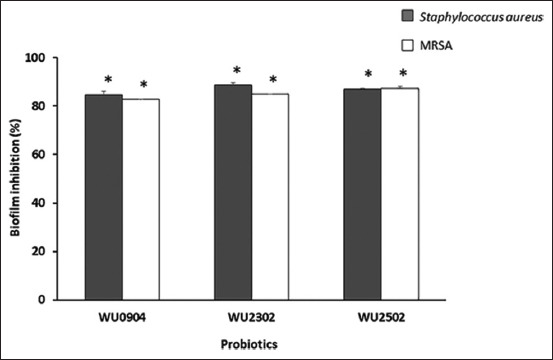
Effects of probiotic supernatants on biofilm formation of *Staphylococcus aureus* ATCC25923 and MRSA. The organism was treated with the supernatants, incubated at 37°C for 24 h. The inhibitory activity was carried out using crystal violet assay. The culture medium was used as a negative control. The relative percentage of the biofilm inhibition (%)=100–[(570treatment/A570control)×100], (*significant difference; p<0.05).

A crystal violet assay investigated the effects of probiotic supernatants on the biofilm formation of *S. aureus* ATCC 25923 and MRSA. The three probiotics’ culture supernatants significantly inhibited biofilm formation (p<0.05), as shown in Figure-4. The results demonstrated that 82.81-87.24% biofilm inhibition was observed after MRSA treatment by the culture supernatants, compared with the negative control. Furthermore, 84.78-88.70% inhibition was detected when *S. aureus* was challenged with the probiotic supernatants.

### Identification of probiotic bacteria

The identification of the selected probiotics was carried out using 16S rRNA sequences. The results demonstrated that the isolate WU 2302 (Accession No. MZ008249) was identified as *Lacticaseibacillus* spp. with 98.82% homology compared to the GenBank database (Table-4). It was also found that the isolates WU 0904 (Accession No. MZ008248) and WU 2502 (Accession No. MZ008274) were identified as *L. paracasei* with 96.30-97.78% similarity. However, the 16S rRNA sequence alignment results indicated that the homology of these bacteria was not 100%, suggesting their status as other distinct strains.

**Table 4:** Identification of three probiotics isolated from Palmyra palm sugar.

16S rRNA Sequences from Isolates	Accession No.	Matches to 16S rRNA Sequences from GenBank Database	Identity (%) with GenBank Database
WU0904	K280749.1	*L. paracasei* strain SMVDUDB1	96.48
	MG590101.1	*L. paracasei* strain HX-3	96.30
WU2302	MN438322.1	*Lacticaseibacillus* spp. strain A2093	99.00
	KX057690.1	*Lacticaseibacillus* *paracasei* strain c46	98.82
WU2502	KY307836.1	*Lacticaseibacillus* *paracasei* strain SWB1	96.38
	MK611262.1	*Lacticaseibacillus* *paracasei* strain 2.0.1	97.78

**Figure-5:**
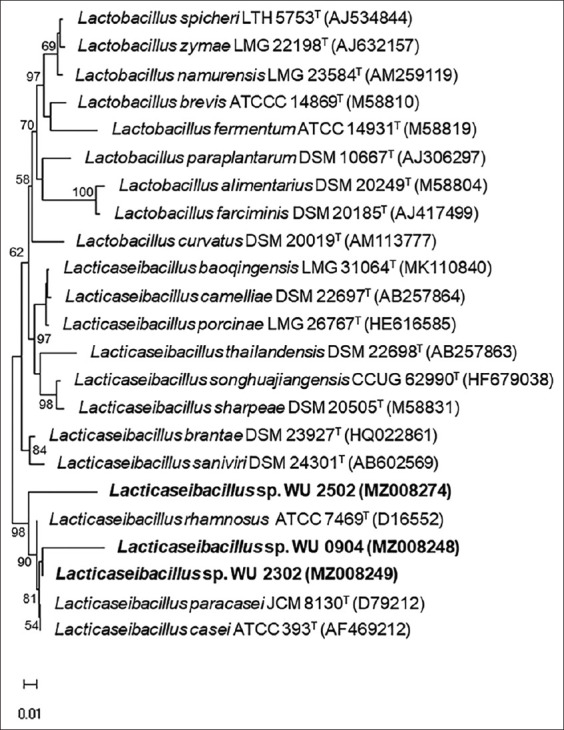
Neighbor-joining phylogenetic tree showing the phylogenetic position of *Lacticaseibacillus* spp. WU 2502, WU 0904, and WU2302 based on 16S rRNA gene sequences. The scale bar indicates 1 nucleotide substitution per 100 nucleotides. Numbers in parentheses indicate accession numbers of 16S rRNA genes from type strains. Bootstrap values over 50% (based on 1000 replications) are shown at the nodes.

As shown in Figure-5, it was observed that the bacteria showed a high similarity of 96-97% with *Lactobacillus* spp., *L. paracasei*, and *Lacticaseibacillus casei*. Thus, the type strains of *Lactobacillus* and *Lacticaseibacillus* genera were selected for phylogenetic tree construction using the neighbor-joining (Figure-5) method, which showed that strains WU 2503, WU 0904, and WU2303 were members of the genus *Lacticaseibacillus*. The close relationships of those strains to *L. paracasei* JCM 8130(T), *L. casei* ATCC 393(T), and *Lacticaseibacillus rhamnosus* ATCC 7469(T) were identified, as shown in Figure-5.

## Discussion

Traditional fermented food is a source of healthy ingredients, as well as beneficial bacteria, including probiotic LAB **[**6**].** In the present study, the lactic acid bacteria isolated from Palmyra palm sugar were chosen and further characterized for their ability to be probiotics.. The three selected isolates of probiotic LAB can produce antimicrobial compounds against MRSA, a new zoonotic pathogen, and food-borne pathogens**.** Threeisolates were identified as *Lacticaseibacillus* spp. with 97% homology compared to the GenBank database. A total of 261 species of the genus *Lactobacillus* have been reported, based on differences at the phenotypic, ecological, and genotypic levels [7]. *L. paracasei*, also known as *L. paracasei*, is part of the microbiota in the human and animal gut. The results of this study suggest that the isolated bacteria are *Lacticaseibacillus* spp. The isolate WU 2302 was identified as *L. paracasei* with 98.82% homology using MALDI-TOF analysis **(**data not shown**).** However, it was noted that the library of the MALDI-TOF analysis instrument had not been updated for the new genus of *Lacticaseibacillus* spp. This genus is well known as part of the normal flora of the gut of humans and animals [6]. The bacterium plays an important role by adhering to the gastrointestinal tract [8], resulting in tract stability and preventing intestinal infections [9]. Our results demonstrated that the isolates were able to adhere to human colon Caco-2 cells**.** It is important to note that the human Caco-2 cell line is commonly used as an *in vitro* model of the intestinal epithelial barrier [24]. The adhesion of probiotics to the colon can prevent the colonization of pathogens**.** The ability of probiotic LAB to act antagonistically against food-borne pathogens indicates their potential as alternatives to chemical drugs**.** It has been reported that probiotic LAB isolated from fermented foods form biofilms on surfaces, which inhibits the colonization of many bacteria, including *L**.** monocytogenes*, *Salmonella* Typhimurium, and *E**.** coli* O157 H7 [10]. Biofilm formation of pathogens is a strategy for surviving and infecting hosts, providing increased resistance to the most commonly used clinical drugs [25]. In this study, the culture supernatants of the three probiotics significantly inhibited biofilm formation by *S**.** aureus* and MRSA**.** The results suggest the potential benefits of the selected probiotic LAB for controlling pathogens**.** *L**.** plantarum* K41 isolated from traditional Sichuan pickles exhibited antibacterial and anti-biofilm activities against *Streptococcus mutans***.** In addition, the probiotic reduced the level of exopolysaccharide production by the pathogen [26]. It has been well-known that *S. aureus*, MRSA, *E. coli* O:157H:7, and *Salmonella* spp. are zoonotic and food-borne pathogens that present the emergence of antibiotic resistance [2,27]**.** *A**.** baumannii* is also classified as a zoonotic pathogen [28]. It has been suggested that the close contact between household pets and people offers favorable conditions for bacterial transmission [28]. In addition, livestock-associated MRSA responsible for human colonization and infection has been reported [29]. However, the microorganisms were inhibited by the supernatant of the isolated probiotics**.**

It is well known that antibiotics are used to treat infectious diseases caused by pathogens. However, antibiotic misuse/non-adherent can result in antibiotic-resistant bacteria [30]. Therefore, the focus has been placed on natural products based on probiotics as an alternative strategy for treating infectious diseases [30]. It is well known that probiotics produce antimicrobial compounds. Our investigation selected LAB that produce antibacterial compounds for further study. To date, antimicrobial compounds produced by *Lactobacillus coryniformis* BCH-4 have been shown to inhibit the growth of *Aspergillus flavus*, a cause of fungal spoilage, in maize grains. In addition, 2-oxopropanoic acid, 2-hydroxypropane-1,2,3-tricarboxylic acid, 2-hydroxybutanedioic acid, 2-hydroxypropanoic acid, propanedioic acid, and butanedioic acid have been identified as bioactive compounds among the antimicrobial compounds isolated from *L*. *coryniformis* [31]. It has been reported that bacteriocin produced by *L**.** paracasei* WX322 severely affected *Pectobacterium carotovorum*, a soft rot pathogen in vegetables [32]. In addition, the probiotic *L*. *paracasei* was found to be a potential barrier to prevent bacterium-associated injury [33]. Specifically, the probiotic could protect host cells from bacterial enterotoxins [33].

The ability to tolerate highly adverse conditions such as those in the gastrointestinal tract, including low pH, and the presence of bile salts and enzymes, is the main factor contributing to the selection of potential probiotic candidates [34]. In this study, all isolates showed relatively strong resistance to pancreatin**.** In addition, 64%–75% resistance to bile salt was detected after the treatment with 3 g/L bile salts. One of the key characteristics for selecting a probiotic candidate is the tolerance to the low pH of the gastric juice in the stomach. Our results showed 80% resistance to a low pH of 3. However, the isolates did not appear to survive after treatment with pepsin or pH 2. Improved probiotic viability under the conditions in the gastrointestinal tract can be achieved by encapsulation. The microencapsulation of *L*. *casei* with calcium alginate and whey protein concentrate was shown to significantly improve the viability of probiotics both in carrier food and under conditions simulating those in the gastrointestinal tract [35]. In addition, a symbiotic, a mixture of probiotics and prebiotics, can enhance the efficacy of probiotics and be used as a technological approach in food applications [36].

## Conclusion

The present study showed the isolation and characterization of probiotics from Palmyra palm sugar, which can produce antimicrobial compounds against MRSA and food-borne pathogens. The three isolates chosen from probiotic LAB were identified as *Lacticaseibacillus* spp., which are capable of producing antimicrobial compounds against important pathogenic bacteria. The isolates were able to tolerate highly adverse conditions such as low pH, and the presence of bile salts and enzymes, as found in the gastrointestinal tract. In addition, the probiotics showed a strong ability to adhere to Caco-2 human colon cells. Moreover, the culture supernatants of the three probiotics significantly inhibited biofilm formation by MRSA. The obtained results strongly suggest that these probiotics are promising candidates for pharmaceutical products.

## Authors’ Contributions

WM, PS, and CR: Conceived and designed the experiments. WM, PS, and CR: Performed the experiments, analyzed, and interpreted the data. WM and CR: Wrote the manuscript. WM and CR: Revised the manuscript. All authors read and approved the final manuscript.
